# Familial hypercholesterolemia – Targeted whole gene sequencing as a diagnostic approach

**DOI:** 10.1016/j.athplu.2024.12.001

**Published:** 2024-12-11

**Authors:** Emma Adolfsson, Nils Johan Fredriksson, Jon Jonasson, Anna Nordenskjöld, Anna Green

**Affiliations:** a Department of Laboratory Medicine, Faculty of Medicine and Health, Örebro University, Örebro, Sweden; b Department of Laboratory Medicine, Örebro University Hospital, Region Örebro County, Sweden; c Department of Cardiology, Faculty of Medicine and Health, Örebro University, Örebro, Sweden

**Keywords:** Familial hypercholesterolemia, Low density lipoprotein receptor, Genetic testing, Sequence analysis, Copy number variation

## Abstract

**Background and aims:**

Familial hypercholesterolemia (FH) and other disorders with similar features are common genetic disorders that remain underdiagnosed and undertreated, due in part to the cost of screening. The aim of this study was to design and implement a whole gene targeted NGS panel for the molecular diagnosis of FH and statin intolerance with an emphasis on high quality variant calling, including copy number analysis.

**Methods:**

A whole gene panel for hybridisation-based short read NGS was designed for the dominant FH-genes low density lipoprotein receptor (*LDLR*), apolipoprotein B (APOB), proproteinconvertas subtilisin/kexin type 9 (PCSK9), apolipoprotein E (APOE) and the recessive FH-genes low density lipoprotein receptor adaptor protein 1 (*LDLRAP1*), ATP binding cassette subfamily member 5/8 (ABCG5/8) and lipase A, lysosomal acid type (*LIPA*), as well as solute carrier organic anion transporter family member 1B1 (*SLCO1B1*), not an FH gene but linked to statin intolerance. Polygenetic risk score markers were also included. The panel was used for screening of a Swedish FH-study population (n = 133).

**Results:**

The panel sequencing resulted in high coverage and confident variant calling of included genes. Known causal variants were found in common dominant FH-genes in 43 % of the cohort. Copy number variants were found in *LDLR* in 10 individuals and a whole gene deletion of *SLCO1B1* in one individual. In addition, coding variants in recessive genes and rare non-coding intronic and untranslated region variants were found in a large proportion of the study individuals highlighting the need for extended gene panels.

**Conclusions:**

This new tool can be used for a comprehensive high-quality molecular genetic analysis according to guidelines for the diagnosis and treatment of FH.

## Highlights

• Design and use of a whole gene targeted NGS panel for molecular diagnosis of FH.
• Confident variant calls, including CNVs, in both coding and non-coding regions.
• Calculation of polygenic risk score and prediction of statin intolerance.
• In a Swedish cohort, n = 133, 43 % were diagnosed with FH due to LDLR or APOB variants.
• CNV calling with NGS eliminated the need for MLPA and found 5 CNVs in 10 individuals.

## 1 Introduction

Familial hypercholesterolemia (FH, OMIM #143890) is an inherited disorder of lipid metabolism, leading to elevated levels of cholesterol and increased risk of coronary artery disease (CAD) and premature myocardial infarction [1]. Depending on the genetic variant or variants causing the disease, FH is categorised as autosomal dominant hypercholesterolemia (ADH), autosomal recessive hypercholesterolemia (ARH) or polygenic hypercholesterolemia [2].

Four dominant genes are associated with the disease: low density lipoprotein receptor (*LDLR)*, apolipoprotein B *(APOB)*, proproteinconvertas subtilisin/kexin type 9 (*PCSK9)* and apolipoprotein E (*APOE)*. *LDLR* pathogenic variants, reducing the efficacy of the receptor, account for >90 % of ADH. *APOB* variants account for 5–7% and *PCSK9* gain-of-function variants and larger duplications for <5 % of ADH [3]. In *APOE,* a pathogenic p.Leu167del variant causes ∼1 % of all FH [4,5].

Additionally, the recessive genes low density lipoprotein receptor adaptor protein (*LDLRAP1)*, ATP binding cassette subfamily member 5/8 (*ABCG5/8)* and lipase A, lysosomal acid type (*LIPA)* can contribute to lipid disorders, predominantly, but not exclusively, by homozygous variants [[5], [6], [7], [8], [9], [10], [11]].

Causal *LDLR* variants for FH include substitutions (hereafter referred to as SNVs), deletions, insertions, duplications and insertion/deletions (hereafter referred to as indels), predominantly in exons but also in introns [12] and UTRs [13,14]. Causal variants in introns and UTRs in other FH-genes have not been extensively studied, but a few variants of unknown significance (VUS) have been reported also for *PCSK9* [14,15] suggesting the importance of screening outside of exons.

Causal single-to multi-exon copy number variants (CNVs) have been reported for *LDLR* [16] and a few duplications have been reported in *APOB* and *PCSK9* [17], but CNVs in other FH-genes remain to a large degree unknown, and are expected to be rare. According to guidelines [18], CNV screening is only required for *LDLR* and the yield is expected to be ∼10 % [19].

Up to 50 % of individuals with clinical FH do not show causative mutations in the most common FH genes (*LDLR*, *APOB*, *PCSK9*) [3]. Broader analysis including the recessive FH genes to capture FH-like disorders, and sequencing outside of known mutational hot spots could give higher yield.

Genetic testing for FH diagnostics can be done using different assays [20]. SNP arrays detect a small number of known SNVs/indels, does not detect CNVs, and will not provide new knowledge in terms of novel genetic mechanisms. In the last 10 years, targeted NGS-based methods have been introduced and taken into clinical practise. The detection rates using NGS-based methods are higher than for SNP arrays [21]. The commercial NGS-based panels commonly targets exons and exon/intron boundaries of *LDLR*, *APOB*, *PCSK9* and *LDLRAP1* [22]*.* Such panels will consequently fail to detect deep intronic- and UTR-variants in these genes as well as variants in other genes possibly linked to FH or lipid disorders. Furthermore, detection of CNVs using NGS data requires high and uniform coverage and is harder for exon only targeted NGS data. To capture CNVs in *LDLR*, SNP arrays or targeted NGS testing are therefore usually combined with multiplex ligation amplification (MLPA) for *LDLR*, increasing the analysis cost. The extensively researched LipidSeq panel [23] targets exons ±250 basepairs of introns and/or UTR in >60 genes, utilizes CNV calling from NGS data, but like whole exome sequencing does not capture deep non-coding variants. Whole genome sequencing could be used for both detection of intronic variants and CNV analysis, but is still, despite reduction in sequencing costs, too expensive for cost effective FH screening.

This study aimed to design and implement a targeted NGS gene panel that enables the detection of all common forms of FH as well as lipid disorders with similar phenotypes. The panel should therefore allow for detection of SNVs/indels and CNVs in genes connected to elevated cholesterol levels. In addition to exons, the panel should cover introns and UTRs to allow for the identification of novel variants. Additionally, the panel should enable identification of polygenic risk markers to further predict disease risk [24] and pharmacogenetic variants to predict drug response to statins [25]. This new genetic testing tool was applied to a Swedish FH-study population.

## 2 Patients and methods

### 2.1 Patient cohort

Patients were recruited 2019–2023 from the cardiology department at Örebro University Hospital, Sweden. Inclusion criteria for participation were a high clinical suspicion of FH and a Dutch Lipid Clinic Network criteria (DLCN) score ≥4. Due to cascade screening of index patients some first-degree relatives were also included in the study (some of which with lower DLCN scores). Written informed consent was required for inclusion. The study was performed in accordance with the Declaration of Helsinki and was approved by the Regional Ethical Review Board of Uppsala (2019–03706/2022-03508-02).

The study cohort consisted of 133 participants, 64 male and 69 female. The average age at inclusion was 51 ± 14 years, range 21–84. Study participants were categorised using DLCN criteria as *Definite FH* (n = 20, 15 %), *Probable FH* (n = 51, 38.3 %), *Possible FH* (n = 57, 42.9 %) and *Unlikely FH* (n = 5, 3.7 %).

### 2.2 Gene panel design

The NGS gene panel was designed for use with Twist Bioscience probe capture hybridisation technology (www.twistbioscience.com). MANE select transcripts encoding whole genes were included for *LDLR*, *APOB*, *PCSK9*, *LDLRAP1*, *APOE, ABCG5*/*8* and *SLCO1B1*. Only exons were included for the gene *LIPA* due to the large size of the gene (Supplemental Table 1). Furthermore, two pharmacogenetic positions linked to statin tolerance were included (Supplemental Table 2).

After the initial design and analysis of 99 participants, an extended updated version of the panel was designed and used for 35 participants. The extended version included more hybridisation probes in *LDLR* UTRs to capture UTR variants, as well as probes for sites used for calculation of weighted polygenic risk score (12 SNPs w-PRS LDL-C) [24] (Supplemental Tables 1 and 2).

### 2.3 Genetic testing

DNA was extracted from 200 μl whole blood using magLEAD® 12gC,Magtration® system, following manufacturer's instructions.

Genomic DNA was analysed by NGS using probe capture hybridisation-based library chemistry from Twist Bioscience with sequencing on Illumina platforms. In short, 50 ng of genomic DNA was used to produce individual libraries using Twist enzymatic fragmentation and hybridisation protocols with Twist universal adapter system [26]. Two different approaches were used; one approach (with the original panel) using short fragments and 2x151 bp paired-end sequencing on MiSeq, n = 98, and the other approach (with the updated panel) using longer fragments and 2x301 bp paired-end sequencing on NextSeq 2000, n = 35. A subset of samples, n = 14, were sequenced using the same source DNA with both approaches (Details are given in Supplemental material).

Processing of FASTQ files, alignment and variant calling was performed using a bcbio bioinformatics pipeline [27] (see Supplemental material for details). CNV calling was done using CNV-Z [28]. All available samples prepared with the same library protocol, hybridised with the same panel and sequenced using the same chemistry were used as reference cohort in the CNV analysis. Small indels <50 bp were assigned as SNVs/indels and not CNVs.

To further characterize and identify breakpoints selected samples with CNVs were analysed using optical genome mapping (OGM) (Saphyr system, BioNano Genomics, San Diego, CA, USA). BioNano Services Lab performed the OGM procedure and data analysis. De novo assembly and variant annotation pipelines were executed on BioNano Solve v3.7. BioNano Access v1.7 were used for CNV reporting and visualisation.

### 2.4 Variant calling validation

SNV and indel calling was validated by comparison with high-confidence variant calls in NA12878 [29]. Sensitivity and positive predictive value (PPV) were calculated for all variant types combined and for SNVs and indels separately. In addition, variants were filtered for overlap with regions known to be problematic for sequencing or variant calling [30,31]. False negative and positive variants were manually inspected in IGV and variants with low coverage (<10X) located in repeats or poly-N regions were considered artefacts and not true false variants. (See Supplemental material for details.)

### 2.5 Variant annotation and pathogenicity assessment

All SNVs/indel variants were imported into QIAGEN QCI-Interpret (version 7.1). Variants were filtered using the gnomAD database version 3.1.2 and variants with a total population variant allele frequency >5 % were removed. Remaining variants were assessed for pathogenicity using the general ACMG guideline for variant pathogenicity assessment [32], and for *LDLR*, specific *LDLR* guidelines from ClinGen [18].

### 2.6 Detection rate and diagnostic yield

The ability to detect genetic variation was evaluated as diagnostic yield in the study population. A diagnosis of monogenic FH was given to study participants with a pathogenic/likely pathogenic variant in a dominant FH-gene, any zygosity, or with a homozygous pathogenic/likely pathogenic variant in a recessive FH-gene.

## 3 Results

### 3.1 Panel coverage and characteristics

Panel coverage is presented in Table 1. All exons in all genes were fully covered with both versions of the panel, with the exception of *SLCO1B1* and the original panel design, where 43 % of samples had an exon coverage lower than 100 % > 30X, 94 % at the lowest. The updated panel and longer sequencing resulted in higher coverage of intronic regions and UTRs for all genes except *SLCO1B1* where intronic coverage deteriorated due to changes in probe placement. The pharmacogenetic markers and SNP w-PRS sites included in respective panel version were all covered above 30X for all samples (For full details, see Supplemental Tables 3–5).

**Table 1:** Gene panel coverage Panel coverage and characteristics for both original and updated gene panel, presented as percentage of bases covered above 30X, total as well as for exons, introns and UTRs separately. All values are mean values based on all sequenced samples.

Gene	Total (%)		Exons (%)		Introns (%)		UTRs (%)	
	Original	Updated	Original	Updated	Original	Updated	Original	Updated
*ABCG5*	95.7	99.8	100	100	95.2	100	100	100
*ABCG8*	95.8	97.6	100	100	95.1	97.1	99.3	100
*APOB*	99.5	100	100	100	99.2	100	100	100
*APOE*	99.0	100	100	100	98.5	100	100	100
*LDLR*	85.7	97.5	100	100	87.7	97.9	42.2	88.6
*LDLRAP1*	99.2	100	100	100	99.1	100	100	100
*LIPA*	14.0	24.6	100	100	8.0	19.4	100	100
*PCSK9*	100	100	100	100	100	100	100	100
*SLCO1B1*	85.9	83.8	99.4	100	85.5	83.4	100	100

Coverage of *LDLR* was variable within the gene due to *Alu*-repeats. *LDLR* has 98 *Alu*-repeats dispersed throughout the introns and *Alu*-repeats in close proximity to each other resulted in drastic reduction or even absence of coverage. However, according to the LOVD LDLR database [33] no known disease-causing variants are located within these “NGS dead zones”. (See Supplemental Fig. 1). Coverage of intronic regions and UTRs in *LDLR* increased with the updated version of the panel and sequencing of longer fragments (on average 97.5 % of *LDLR* had >30X coverage compared to 85.7 % using the original panel/short sequencing) (Supplemental Tables 3 and 4).

In addition to overall increased coverage, sequencing of longer fragments resulted in improved mapping and improved CNV calling (see Supplemental Table 6 and Supplemental Fig. 2 for details).

### 3.2 Validation of called variants

Called SNVs and indels showed high concordance with the truth set for NA12878 [26]. After adjustment for problematic regions and manual removal of artefacts, PPV was 100 % for both indels and SNVs for both the original and updated panel. Sensitivity for SNVs was 98.4 % (original panel) and 96.7 % (updated panel) and for indels 97.7 % (original panel) and 94.3 % (updated panel), see Supplemental Table 7 for details.

False negative variants were all due to lack of coverage, a few in the *LDLR* NGS dead zones mentioned above and most in intronic regions of *SLCO1B1*, where coverage was lower in the updated panel. *SLCO1B*1 is not an FH gene and statin intolerance is assessed by exonic *SLCO1B1* variants. Therefore, although included in the panel, *SLCO1B1* introns are more relevant for research purposes than of clinical importance. Excluding *SLCO1B1* introns from the validation reduces the number of false negative variants found, and specifically reduces false negative indels to zero for the updated panel, increasing the sensitivity from 94.3 % to 100 %. Excluding *SCLO1B1* introns from the validation would therefore result in both sensitivity and PPV above the threshold accepted, 95 %, for currently applied methods in the clinic, both for the original and updated panel.

All called CNVs in *LDLR* were compared and in agreement with previous results from MLPA Salsa P062 *LDLR* kit, McHolland.

### 3.3 Outcome of genetic screening

#### 3.3.1 Findings in dominant genes; LDLR, APOB, PCSK9

**Fig. 1:**
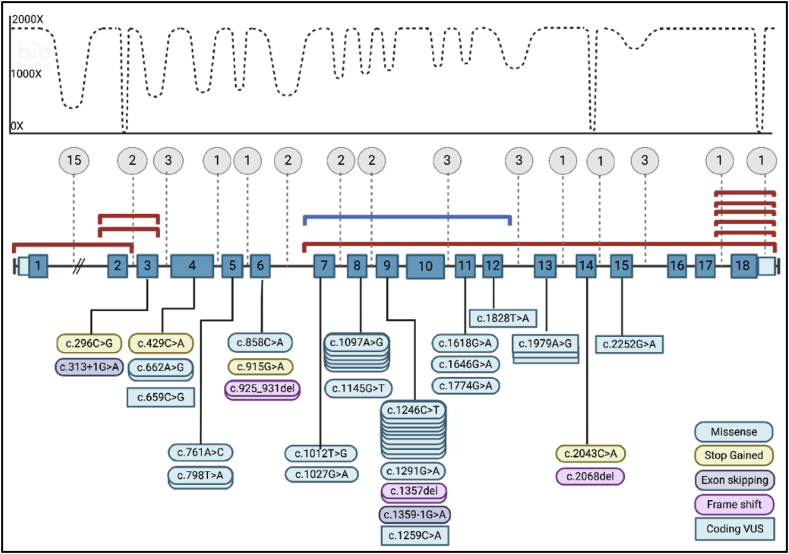
LDLR *variants detected in the study cohort.* Disease-causing SNVs/indels were found in exons and splice sites and shown as rounded boxes. Coding VUS, all missense, are shown in squared boxes. The number of boxes for each variant indicates variant frequency in the study population. Copy number variants are represented as brackets above the genes, encompassing the exons deleted (blue bracket) or duplicated (red brackets). Non-coding VUS are shown in grey circles with the number of VUS in each intron or UTR given. General depth of coverage is illustrated with areas of missing coverage in intron 2, intron 14 and 3′UTR for samples analysed on NextSeq2000. Created with BioRender.com.

Variants in *LDLR* were the most frequent; 27 pathogenic/likely pathogenic variants and 5 coding variants of unknown significance (VUS) with a strong connection to FH were found. Missense variants (n = 18) were the most frequent followed by nonsense (n = 4), frameshift (n = 3) and splice site variants (n = 2). Additionally, a large number of rare non-coding VUS in introns and 3′UTRs were detected (see Fig. 1 and Supplemental Table 8).

In *APOB* two pathogenic heterozygous missense mutations located in exon 26, known to harbour variants resulting in FH, were detected, as well as coding (n = 7) and non-coding (n = 21) VUS. Four coding VUS in *PCSK9* were detected. The c.385G > A, p.Asp129Asn, is a rare missense gain-of-function variant described previously as disease-causing in a Scandinavian cohort [34] and more likely to contribute to disease. Additionally, 20 non-coding VUS were identified (see Supplemental Table 8).

**Fig. 2:**
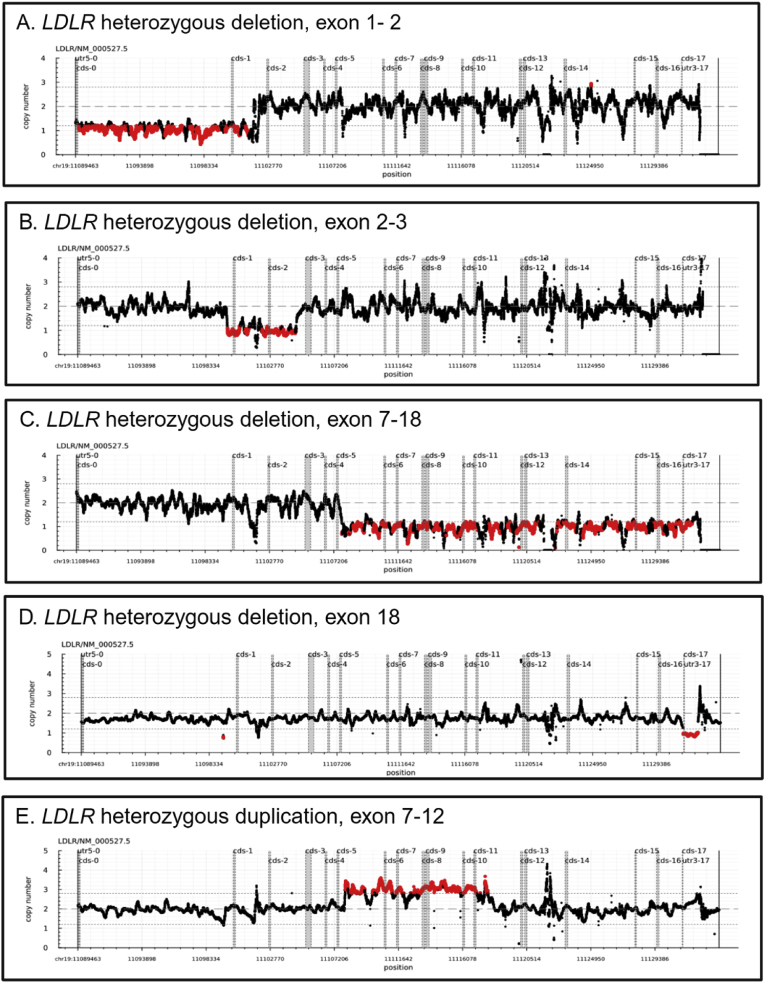
LDLR *CNVs detected in the cohort.* From top to bottom; A. heterozygous deletion exon 1–2, B. heterozygous deletion exon 2–3, C. heterozygous deletion exon 7–18, D. heterozygous deletion exon 18, E. heterozygous duplication exon 7–12. CNVs are called using CNV-Z from NGS data. Each graph shows the calculated copy number for each position. Positions with a significantly higher coverage (duplications) or lower coverage (deletions) than expected are displayed in red.

Five different CNVs in *LDLR* were detected; four heterozygous deletions and one heterozygous duplication, see Fig. 2. In total, causal CNVs in *LDLR* were detected in 10 individuals (7.5 %). Deletion of exon 1–2 was identified in one study subject. Optical genome mapping (OGM) results indicated that the 18.2 kB aberration expands upstream into *SMARCA4* gene, and the deletion of the first two exons is predicted to result in no *LDLR* protein [35].

Deletion of exon 2–3 was identified in two related subjects. The breakpoints were visible in IGV, and the size in both cases was 5.5 kB. Due to polymorphisms in the OGM sequence recognition motifs, OGM analysis falsely determined the deletion to be much larger, demonstrating a limitation in the use of OGM to characterize small CNVs. The deletion results in reduction of *LDLR* efficacy to 70 %, resulting in a mild FH phenotype [36].

The exon 7–18 deletion was found in one individual. The deletion was 127.9 kB and encompasses other genes downstream of *LDLR*.as determined with OGM analysis. According to LOVD LDLR [33] the deletion results in a truncated protein.

Duplication of exon 7–12 was identified in one study subject. This duplication was not analysed using OGM. This 6.3 kB duplication has previously been reported in Danish and Taiwanese populations [37,38] and is predicted to result in a larger *LDLR* protein with a shorter half-life, resulting in increased LDL-C levels [38].

The most frequent CNV was the exon 18 deletion, identified in five related subjects. This deletion was not analysed using OGM, but using IGV breakpoints were identified, and the size estimated as 1.5 kB. To our knowledge, the genotype- and phenotype relationship for this deletion has not been characterised.

#### 3.3.2 Findings in recessives genes; LDRAP1, ABCG5/8, LIPA

No homozygous variants were detected in the recessive genes, i.e. no autosomal recessive hypercholesterolemia (*LDLRAP1*), siterolemia (*ABCG5/8*) or lysosomal acid lipase deficiency (*LIPA*). Coding heterozygous variants classified as VUS were detected in *ABCG5* (n = 3), *ABCG8* (n = 5), *LDLRAP1* (n = 2) and *LIPA* (n = 2), see Table 2. Rare non-coding variants found in *ABCG8*, *LIPA* and *LDLRAP1* are listed in Supplemental Table 9.

**Table 2:** Coding and splice-site SNVs/indels detected in the FH-cohort. *Coding and splice-site SNV/indels found in the study population, presented per gene and with cohort frequency. Additionally, heterozygous VUS in recessive genes are presented. Variants are listed with c.position, p.position, type of variant, effect on code, assessed pathogenicity, and no. of observations in cohort. Transcripts as follows:* ABCG5 *NM_022436.3,* ABCG8 *NM_022437.3,* APOB *NM_000384.3,* APOE *NM_000041.4,* LDLR *NM_000527.5,* LDLRAP1 *NM_015627.3,* LIPA *NM_000235.4,* PCSK9 *NM_174936.4,* SLCO1B1 NM_006446.5

Gene	Mode of inheritance	Location	c.position	p.position	Type	Effect	Assessment	No.
*LDLR*	Dominant	Exon 9	c.1246C > T	p.Arg416Trp	Substitution	Missense	Pathogenic	10
*LDLR*	Dominant	Exon 8	c.1097A > G	p.Gln366Arg	Substitution	Missense	Likely pathogenic	6
*LDLR*	Dominant	Exon 5	c.798T > A	p.Asp266Glu	Substitution	Missense	Pathogenic	2
*LDLR*	Dominant	Exon 4	c.662A > G	p.Asp221Gly	Substitution	Missense	Pathogenic	2
*LDLR*	Dominant	Exon 6	c.925_931del	p.Pro309Lysfs∗59	Deletion	Frameshift	Pathogenic	2
*LDLR*	Dominant	Exon 9	c.1357delA	p.Ser453Alafs∗54	Deletion	Frameshift	Likely pathogenic	2
*LDLR*	Dominant	Exon 7	c.1027G > A	p.Gly343Ser	Substitution	Missense	Pathogenic	1
*LDLR*	Dominant	Exon 3	c.313+1G > A		Substitution	Exon Skip	Pathogenic	1
*LDLR*	Dominant	Exon 3	c.296C > G	p.Ser99∗	Substitution	Stop Gain	Pathogenic	1
*LDLR*	Dominant	Exon 4	c.429C > A	p.Cys143∗	Substitution	Stop Gain	Pathogenic	1
*LDLR*	Dominant	Exon 5	c.761A > C	p.Gln254Pro	Substitution	Missense	Likely pathogenic	1
*LDLR*	Dominant	Exon 6	c.858C > A	p.Ser286Arg	Substitution	Missense	Likely pathogenic	1
*LDLR*	Dominant	Exon 6	c.915G > A	p.Trp305∗	Substitution	Stop Gain	Pathogenic	1
*LDLR*	Dominant	Exon 7	c.1012T > G	p.Cys338G	Substitution	Missense	Likely pathogenic	1
*LDLR*	Dominant	Exon 8	c.1145G > T	p.Gly382Val	Substitution	Missense	Likely pathogenic	1
*LDLR*	Dominant	Exon 9	c.1291G > A	p.Ala431Thr	Substitution	Missense	Pathogenic	1
*LDLR*	Dominant	Exon 11	c.1618G > A	p.Ala540Thr	Substitution	Missense	Likely pathogenic	1
*LDLR*	Dominant	Exon 11	c.1646G > A	p.Gly549Asp	Substitution	Missense	Pathogenic	1
*LDLR*	Dominant	Exon 12	c.1774G > A	p.Gly592Arg	Substitution	Missense	Likely pathogenic	1
*LDLR*	Dominant	Exon 14	c.2043C > A	p.Cys681∗	Substitution	Stop Gain	Pathogenic	1
*LDLR*	Dominant	Exon 14	c.2068del	p.His690fs∗19	Deletion	Frameshift	Pathogenic	1
*LDLR*	Dominant	Exon 10	c.1359-1G > A		Substitution	Exon skip	Pathogenic	1
*LDLR*	Dominant	Exon 4	c.659C > G	p.Pro220Arg	Substitution	Missense	VUS	1
*LDLR*	Dominant	Exon 9	c.1259C > A	p.Thr420Asn	Substitution	Missense	VUS	1
*LDLR*	Dominant	Exon 12	c.1828T > A	p.Ser610Thr	Substitution	Missense	VUS	1
*LDLR*	Dominant	Exon 13	c.1979A > G	p.Gln660Arg	Substitution	Missense	VUS	3
*LDLR*	Dominant	Exon 15	c.2252G > A	p.Arg751Gln	Substitution	Missense	VUS	1
*APOB*	Dominant	Exon 26	c.10580G > A	p.Arg3527Gln	Substitution	Missense	Pathogenic	6
*APOB*	Dominant	Exon 26	c.10579C > T	p.Arg3527Trp	Substitution	Missense	Pathogenic	1
*APOB*	Dominant	Exon 3	c.218C > A	p.Ala73Asp	Substitution	Missense	VUS	2
*APOB*	Dominant	Exon 10	c.1207G > A	p.Ala403Thr	Substitution	Missense	VUS	1
*APOB*	Dominant	Exon 18	c.2630C > T	p.Pro877Leu	Substitution	Missense	VUS	1
*APOB*	Dominant	Exon 24	c.3724T > A	p.Ser1242Thr	Substitution	Missense	VUS	1
*APOB*	Dominant	Exon 26	c.10015T > C	p.Tyr3339His	Substitution	Missense	VUS	1
*APOB*	Dominant	Exon 26	c.10117G > C	p.Val3373Leu	Substitution	Missense	VUS	1
*APOB*	Dominant	Exon 26	c.10370C > G	p.Ser3457Cys	Substitution	Missense	VUS	1
*APOB*	Dominant	Exon 29	c.12536C > T	p.Thr4179Ile	Substitution	Missense	VUS	1
*PCSK9*	Dominant	Exon 2	c.385G > A	p.Asp129Asn	Substitution	Missense	VUS	1
*PCSK9*	Dominant	Exon 7	c.1030C > A	p.Gln344Lys	Substitution	Missense	VUS	1
*PCSK9*	Dominant	Exon 7	c.1152G > C	p.Gly384 =	Substitution	Silent	VUS	1
*PCSK9*	Dominant	Exon 9	c.1487G > A	p.Arg496Gln	Substitution	Missense	VUS	1
*ABCG5*	Recessive	Exon 3	c.392A > G	p.Tyr131Cys	Substitution	Missense	VUS	1
*ABCG5*	Recessive	Exon 8	c.940C > T	p.Arg314Trp	Substitution	Missense	VUS	1
*ABCG5*	Recessive	Exon 9	c.1166G > A	p. Arg389His	Substitution	Missense	VUS	1
*ABCG8*	Recessive	Exon 5	c.613G > C	p.Val205Leu	Substitution	Missense	VUS	1
*ABCG8*	Recessive	Exon 5	c.628G > T	p.Val210Leu	Substitution	Missense	VUS	1
*ABCG8*	Recessive	Exon 9	c.1160C > T	p.Pro387Leu	Substitution	Missense	VUS	1
*ABCG8*	Recessive	Exon 10	c.1412G > T	p.Cys471Phe	Substitution	Missense	VUS	1
*ABCG8*	Recessive	Exon 10	c.1436A > G	p.Tyr479Cys	Substitution	Missense	VUS	1
*LDLRAP1*	Recessive	Exon 2	c.105G > A	p.Trp35∗	Substitution	Stop Gain	Pathogenic	1
*LDLRAP1*	Recessive	Exon 3	c.284G > A	p.Arg95Gln	Substitution	Missense	VUS	1
*LDLRAP1*	Recessive	Exon 4	c.451C > T	p.Arg151Trp	Substitution	Missense	VUS	1
*LIPA*	Recessive	Exon 1	c.4A > C	p.Lys2Gln	Substitution	Missense	VUS	1
*LIPA*	Recessive	Exon 2	c.74A > T	p.Lys25Ile	Substitution	Missense	VUS	1

#### 3.3.3 Recurring disease-causing variants

All causal variants were found in dominant genes and a few recurring variants in *LDLR* and *APOB* and accounted for the majority of causal findings. As expected, variants in *LDLR* were in absolute majority. The most frequent variant, *LDLR* p.Arg416Trp was found in ten individuals, followed by *APOB* p.Arg3527Gln and *LDLR* p.Gln366Arg, both detected in six study subjects each (see Table 2).

#### 3.3.4 Detection rate and diagnostic yield

In total, 57 study participants of 133 received a molecular monogenic diagnosis of FH due to presence of causal variants in *LDLR* or *APOB*, corresponding to 42.8 %. The majority of these individuals belonged to DLCN category *Definitive* or *Probable* FH (35 of 71, 49.3 %), however, also individuals with *Possible* or *Unlikely* FH carried disease-causing variants, although to a lesser extent (22 of 62, 35.5 %). SNVs/indels accounted for 82.5 % of the findings, and CNVs in *LDLR* accounted for 17.5 %.

### 3.4 Findings in *SLCO1B1* and pharmacogenetic markers

**Fig. 3:**
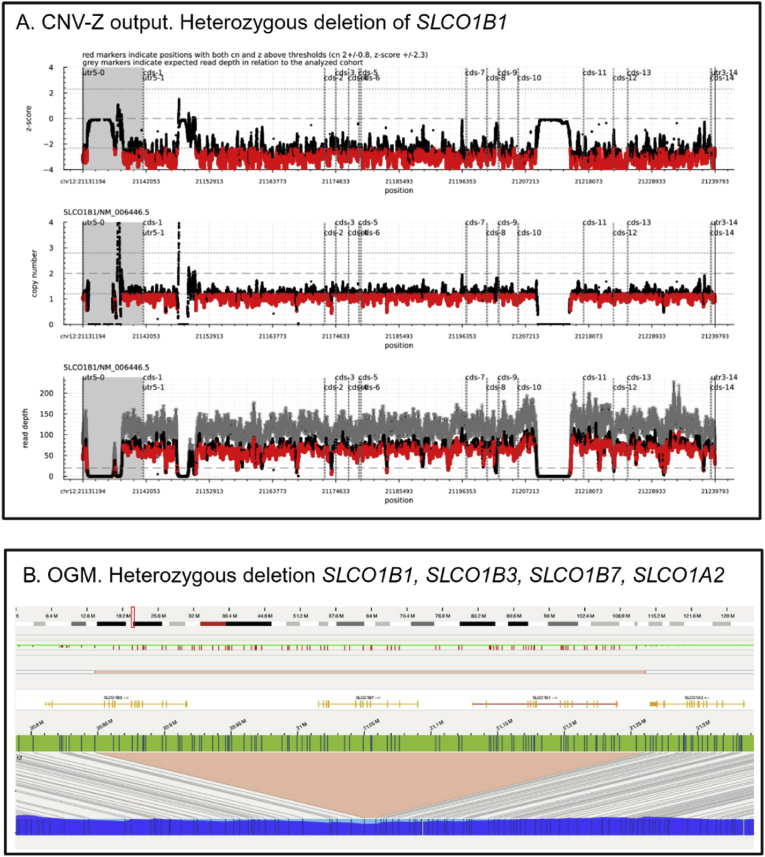
Whole-gene heterozygous deletion of SLCO1B1 found in one individual. *CNV-Z visualisation output for SLCO1B1. The copy number, presented in the middle graph, is around one for the whole gene, indicating a heterozygous deletion. The top graph shows a low z-score, indicating significance. Observed read depth is shown in the bottom graph, with expected read depth, in comparison with the reference cohort, in grey. Coverage in affected sample is roughly half of normal reference cohort. In all three graphs red markers indicate positions with both CN- and z values above thresholds (CN= ±0.8, z= ±2.3). As seen in the bottom graph, parts of the gene lack coverage. B. OGM depicts identified copy number variant in the genomic region containing* SLCO1B1 *as well as* SLCO1A2*,* SLCO1B7 *and* SLCO1B3*. The size of the deletion was estimated to be 404.*6 kB*.*

CNV-analysis of *SLCO1B1* revealed a whole gene heterozygous deletion of *SLCO1B1* (Fig. 3 top). Breakpoints could not be determined using the panel data. OGM analysis confirmed the finding and showed that the deletion expanded upstream and downstream with an estimated size of 404.k kB (Fig. 3 bottom).

In regard to pharmacogenetic markers, 31 individuals had the rs4149056 variant in *SLCO1B1* (heterozygous or homozygous) and 35 individuals had a heterozygous rs2231142 variant in *ABCG2* (Supplemental Table 10).

### 3.5 Findings in polygenic risk score markers

Polygenic risk score markers were only included in the updated version of the panel. In total, 10 of 35 presented with w-PRS LDL-C at or above the eight decile computed according to Ref. [20]. Three of these had both a monogenic and a polygenic component, and seven had just the polygenic components.

## 4 Discussion

In this study, a whole gene NGS panel for FH and similar lipid disorders was designed and applied to a Swedish FH cohort. The panel was based on Twist hybridisation-based chemistry known to give high coverage and uniformity [39]. The panel included whole genes instead of only coding regions to enhance CNV detection and to enable the detection of variants in introns and UTRs not yet linked to FH. Results showed that both small variants, SNVs and indels, and CNVs could be analysed with confidence using this panel.

The purpose of the study was to demonstrate diagnostic screening method for FH for routine clinical laboratories that could also explore genetic variation and in lipid disorders beyond FH. FH is underdiagnosed and having FH increases an individual's risk of cardiovascular disease due to lifelong exposure to elevated cholesterol. Extensive genetic screening with cascade screening for relatives has been deemed cost-effective on the population-wide level if done early in patients' lives [40].

Screening of the cohort resulted in a molecular monogenetic diagnosis for 57 of 133 participants, where casual variants were found in *LDLR* and *APOB.* These 57 were from all DLCN categories, from definitive to unlikely FH. The detection rate of 42.8 % is in line with the detection rate of 46 % reported for LipidSeq for FH [41].

Ten of the 57 molecular diagnoses were based on causal CNVs in *LDLR,* highlighting the importance of CNV detection in FH-screening. CNV detection can be challenging for various reasons. MLPA, which is commonly used, produces easily interpreted results, given high-quality DNA and sufficient high-quality reference samples. However, the MLPA analysis is limited to *LDLR,* requires separate equipment, may miss smaller aberrations and is costly. The results of this study show that short-read whole gene sequencing data is an alternative to MLPA. The CNV-Z tool used has been validated and confirmed to be accurate for *LDLR* [28] and the identified *LDLR* CNVs in this study were all in agreement with previous analyses with MLPA. As with MLPA, there are also challenges with CNV calling from short read sequencing data. Both MLPA and CNV-Z depend on reference samples and if several individuals in the same analysis are carrying the same aberration, the precision may be negatively affected. The CNV calling with short-read targeted NGS data also requires high coverage and detection and precision is affected by low or lack of coverage, which was seen in the *LDLR* exon 1–2 deletion found in this study. Exon 2 of *LDLR* is flanked by *Alu*-repeats resulting in low or no coverage, and the breakpoint of the deletion could therefore not be determined. Performance may also be affected by sequencing fragment lengths. In this study sequencing was done with two different fragment lengths, and the longer reads improved the performance of CNV-Z with fewer false positives and fewer false negatives. However, while MPLPA is limited to *LDLR*, short read whole gene sequencing allow for the detection of CNVs in any gene included in the panel. Although no CNVs in FH-related genes were discovered in this relatively small cohort, the importance of CNVs in other FH-related genes is currently unknown and has not been investigated in larger cohorts.

Several VUS, both in dominant and recessive genes, were found in participants from all DLCN categories. Rare non-coding deep intronic and UTR variants were found in both dominant genes (Supplemental Table 8) and recessive genes (Supplemental Table 9) in participants without causal variants. Emerging evidence suggests that these types of intronic variants in *LDLR* and *PCSK9* modulates lipid levels [[13], [14], [15]]. The presence of rare non-coding variants in patients with possible FH highlights the importance of broader genetic panels and whole gene sequencing in searching for novel genetic mechanisms to explain lipid disorders.

Screening with pharmacogenetic marker sites resulted in an expected ratio of participants with a predicted altered statin metabolism [42]. In addition, the CNV analysis revealed a *SLCO1B1* deletion. *SLCO1B1* is a major gene for statin intolerance and increasing number of variants have been connected to side effects like myopathy [42]. The significance of this CNV in regard to statin response is to our knowledge unknown at present.

Additional elements to the panel design included SNPs for w-PRS. The panel can therefore be used to detect both types of FH; monogenic and polygenic. Several studies [43,44] show that 12 SNP w-PRS is associated with a risk for high LDL-C and cardiovascular disease (CVD), and in addition high cholesterol can have both a monogenic and a polygenic component that interact. Patients with both monogenic FH and high w-PRS are at greatest risk of CVD [45]. As the markers were added to the second version of the gene panel, only a subset of study participants were screened for w-PRS markers, but results were in the expected range when compared to other studies [24,46].

### 4.1 Strengths and limitations

The targeted whole gene design has several strengths. Targeting whole genes enables the detection of CNVs and allows for non-coding regions to be explored, enabling detection of a broad range of variants relevant to FH, but with a lower sequencing cost than complete WGS. However, a limitation compared to WGS and WES is the inability to expand the analysis to genes not included in the panel, if no genetic variants are detected, as can be done with WES and WGS by step-wise filtering. Redesign and revalidation of the panel would be necessary as new knowledge emerges and previously analysed samples could not be re-evaluated without re-sequencing.

Additional limitations to this study is the small and heterogenous study population and the usage of two versions of the gene panel, limiting the number of cases *per* panel version even more.

Finally, although the whole gene design is regarded as beneficial, as it strengthens the CNV analysis and allows for the exploration of introns and UTR regions, including whole genes in the screening for genetic causes of FH may lead to findings of rare non-coding variants for which little functional evidence exists, making variant interpretation difficult and time-consuming.

## 5 Conclusion

Whole gene sequencing as a diagnostic approach for genetic testing of FH and other lipid disorders has the benefit of enabling both SNVs/indels and CNV detection from the same data set. By including pharmacogenetic and polygenic risk markers, a comprehensive screening and research method can contribute to a high diagnostic yield and new knowledge about these complex genetic disorders.

## CRediT author statement

AN: Conceptualization, Resources, Data curation, Supervision. AG: Conceptualization, Methodology, Formal analysis, Validation, Writing – review and editing, Supervision, Funding acquisition. JJ: software. NJF: Software, Formal analysis, Data curation, Writing – review and editing. EA: Investigation, Formal analysis, Data curation, Writing – initial draft, Writing – review and editing, Visualisation, Project administration, Funding acquisition.

All authors read and approved the final version.

## Financial support

The study received grants from Örebro County Research Committee. EA received funding from ALF Funding Region Örebro County.

## Declaration of competing interest

The authors declare that they have no known competing financial interests or personal relationships that could have appeared to influence the work reported in this paper.
