# Supplementary material for: Familial hypercholesterolemia – Targeted whole gene sequencing as a diagnostic approach
Source: Atheroscler Plus. 2024 Dec 11;59:1–9. doi: 10.1016/j.athplu.2024.12.001 (PMC11719334; doi:10.1016/j.athplu.2024.12.001)
Supplement: Multimedia component 1 [file mmc1.docx]

# Supplemental material to “Familial hypercholesterolemia – targeted whole gene sequencing as a diagnostic approach”

## NGS gene panel ordering info:

Gene panels can be ordered through Twist Bioscience using the following ordering info and approval from AG:

- Original NGS FH panel: **“**TE-96267699_hg38”
- Updated NGS FH panel: “TE-95144787_LowStringency”

For content, see Supplemental Table 1 and 2.

## Library preparation and sequencing approaches

Library enzymatic fragmentation and hybridisation was done using Twist chemistry and Twist protocols for enzymatic fragmentation and hybridisation with Twist universal adapter system (https://www.twistbioscience.com/resources/protocol/enzymatic-fragmentation-and-twist-universal-adapter-system).

To obtain short fragments, enzymatic fragmentation was performed at +37° C for 8 minutes, followed by 10 cycles of pre-hybridisation amplification. Eight samples were pooled in equimolar content to a final mass of 1500 ng, and hybridised overnight with the original gene panel, followed by post-hybridisation amplification for 13 cycles. Quality controlling steps included DNA quantification measurements using Qubit 2.0 (ThermoFisher, Waltham, MA) and fragment length analysis using Tapestation D1000 Screen Tape (Agilent Technologies, Santa Clara, CA) of both individual libraries and the final pool. Two pools, i.e. 16 samples, were sequenced on MiSeq (Illumina, San Diego, CA) using 2x151 bp paired-end chemistry, with 6 pM input DNA. In total 98 samples were analysed using panel 1 during 2019-2021.

To obtain long fragments, the enzymatic fragmentation was performed at +30° C for 7 minutes, followed by 8 cycles of pre-hybridisation amplification. The same hybridisation protocol as for short fragments was used but with the updated gene panel. The obtained pools were sequenced on NextSeq2000, 2x301 bp paired-end chemistry, using 1pM input DNA. A total of 35 samples were analysed using this approach during 2022-2023.

To allow for comparisons the Coriell Platinum genome sample NA12878 [1] were analysed using both approaches.

## Bioinformatic pipeline

bcbio-nextgen 1.1.5 was used for processing of FASTQ files, alignment and variant calling. Reads were aligned to human genome build 38 (GRCh38) using bwa version 0.7.17 [2]. SAMtools version 0.1.9 [3] was used to sort and index BAM files. SNV/indel variant calling was performed using three different callers, GATK version 3.8 [4], SAMtools version 0.1.9 [5] and freebayes version 1.10.46 [6]. Ensemble multiVCF files were obtained including all calls where at least two of the callers had detected a variant. Individual VCF files containing only non-reference calls were obtained using VCFtools version 0.1.16 [7]. Sequencing quality control was performed with fastqc version 0.11.8, picard version 2.20.2, mosdepth version 0.3.3 and samtools version 0.1.9 and inspected using multi-QC version 1.14 [8].

## CNV-calling

CNV calling was done using CNV-Z [9]. CNV plots were generated using dedicated julia scripts (available upon request).

Thresholds for reporting CNVs were set as ±2.3 (z-score), ±0.8 (copy number threshold), corresponding to copy number 1.2 for deletions and 2.8 for duplications.

CNV calling from longer NGS reads with CNV-Z resulted in both fewer false positives and fewer false negatives also in genes other than *LDLR* (Supplemental Figure 2).

## Variant calling validation

Platinum genomes truthset VCF and region bed file, version 2017-1.0, were downloaded from sources listed at https://github.com/Illumina/PlatinumGenomes.

Region bed files for regions problematic for sequencing or variant calling as described in [10] were downloaded from <https://ftp-trace.ncbi.nlm.nih.gov/ReferenceSamples/giab/release/genome-stratifications/>. Regions included were tandem repeats and homopolymers (GRCh38_AllTandemRepeatsandHomopolymers_slop5.bed), low mappability regions (GRCh38_lowmappabilityall.bed), problematic promoter regions (GRCh38_BadPromoters.bed) and other difficult regions (GRCh38_allOtherDifficultregions.bed). In addition, regions described as problematic for alignment using short reads [11] were also included.

The truthset VCF was filtered to only include variants in regions corresponding to whole genes *ABCG5*, *ABCG8*, *APOB*, *APOE*, *LDLR*, *LDLRAP1*, *PCSK9* and *SLCO1B1* (MANE Select transcripts) and coding exons of *LIPA*, including +/- 40bp. VCFs for NA12878 obtained in this study were filtered to only include variants in confident regions, as defined in the platinum genomes data set. All VCFs were also filtered to only include variants not in problematic regions. VCF files were filtered and compared using VCFtools, version 0.1.16 [7].

Sensitivity and positive predictive value (PPV) were calculated using the following definitions:

- true positive variants = variants in the same position and with the same alternative base value in both truth set VCF and study VCF
- mismatching variants = variants in the same position in the truth set VCF and the study VCF, but with different alternative base values
- false positive variants = variants only in study VCF + mismatching variants
- false negative variants = variants only in truth set VCF + mismatching variants

Variants found only in the truth set or only in the study VCFs were inspected manually using IGV, version 2.11.9 [12] to determine the cause of the discrepancy. Variants located in poorly covered poly-N regions or within repeats were categorised as artefacts, whereas variants not detected due to lack of coverage after targeted NGS, were considered false negatives, since the comparison aimed to test the performance of the panel in the complete genes. All false negative variants were located in introns in *SLCO1B1* and intron 14 and 3´UTR of *LDLR*, regions not known to harbour pathogenic variants of relevance for lipid disorders. The results of the comparison are summarised in Supplemental table 7. PPV was 1 for both approaches after filtering and removal of artefacts, whereas sensitivity was lower using the updated version of the panel due to slightly higher number of false negatives, caused by lack of coverage in *SLCO1B1* intronic regions.

***Supplemental Table 1. Gene content of in-house gene panels for FH diagnostics.***

*Table shows abbreviated gene name, HGNC approved name, part of the gene covered by design, transcript, and genomic location on GRCh38 for the original and updated gene panel.*

| **Gene** | **HGNC approved name** | **Coverage** | **Transcript** | **Original panel**  **GRCh38 location** | **Updated panel**  **GRCh38 location** |
| --- | --- | --- | --- | --- | --- |
| *ABCG5* | ATP binding cassette subfamily G member 5 | Complete | NM_022436.3 | 2:43806154– 2:43838840 | 2:43806153– 2:43838840 |
| *ABCG8* | ATP binding cassette subfamily G member 8 | Complete | NM_022437.3 | 2:43831942– 2:43880051 | 2:43831942– 2:43880051 |
| *APOB* | Apolipoprotein B | Complete | NM_000384.3 | 2:21001429– 2:21044073 | 2:21001428– 2:21044073 |
| *APOE* | Apolipoprotein E | Complete | NM_000041.4 | 19:44905749– 19:44909395 | 19:44905790– 19:44909393 |
| *LDLR* | Low density lipoprotein receptor | Complete | NM_000527.5 | 19:11089362– 19:11133830 | 19:11087883– 19:11133857 |
| *LDLRAP1* | Low density lipoprotein receptor adaptor protein 1 | Complete | NM_015627.3 | 1:25543580– 1:25590400 | 1:25543580– 1:25590400 |
| *LIPA* | Lipase A, lysosomal acid type | Exons only | NM_000235.4 | 10:89213569– 10:89252039 | 10:89213568– 10:89414557 |
| *PCSK9* | Proprotein convertase subtilisin/kexin type 9 | Complete | NM_174936.4 | 1:55039476– 1:55064853 | 1:55038976– 1:55064852 |
| *SLCO1B1* | Solute carrier organic anion transporter family member 1B1 | Complete | NM_006446.5 | 12:21131194– 12:21239796 | 12:21131193– 12:21239796 |

***Supplemental Table 2.*** ***Additional contents of the in-house gene panels for FH diagnostics.***

*12 SNPs weighted LDL-C polygenic risk markers [13] and SNPs for statins clearance/statin response with clinical evidence (Pharm GKB). Some markers were included in both version of the gene panel (“both”) whereas some were included only in the updated panel, gene panel version 2 (“updated”).*

| **RSID*** | **Gene** | **HGVS** | **GRCh38 location** | **Panels** |
| --- | --- | --- | --- | --- |
| **Markers for LDL-C polygenic risk score, 12 w-PRS** | | | |  |
| rs2479409 | *PCSK9* | NC_000001.10:g.55504650G>A | 1: 55038977 | Updated |
| rs4299376 | *ABCG8* | NC_000002.12:g.43845437G>T | 2:43845437 | Both |
| rs1367117 | *APOB* | NC_000002.12:g.21041028G>A | 2:21041028 | Both |
| rs6511720 | *LDLR* | NC_000019.10:g.11091630G>T | 19:11091630 | Both |
| rs629301 | *CELSR2* | NC_000001.10:g.109818306G>T | 1:109275684 | Updated |
| rs429358 | *APOE* | NC_000019.10:g.44908684T>C | 19:44908684 | Both |
| rs7412 | *APOE* | NC_000019.10:g.44908822C>T | 19:44908822 | Both |
| rs1564348 | *SLC22A1* | NC_000006.12:g.160157828T>C | 6:160157828 | Updated |
| rs1800562 | *HFE* | NC_000006.12:g.26092913G>A | 6:26092913 | Updated |
| rs3757354 | *MYLIP* | NC_000006.12:g.16127176C>T | 6:16127176 | Updated |
| rs11220462 | *ST3GAL4* | NC_000011.10:g.126374057G>A | 11:126374057 | Updated |
| rs8017377 | *NYNRIN* | NC_000014.9:g.24414681G>A | 14:24414681 | Updated |
| **Markers for statin clearance/statin response** | | | |  |
| [rs4149056](https://www.pharmgkb.org/variant/PA166155341) | *SLCO1B1* | NC_000012.12:g.21178615T>C | 12:21178615 | Both |
| [rs2231142](https://www.pharmgkb.org/variant/PA166156544) | *ABCG2* | NC_000004.12:g.88131171G>T | 4:88131171 | Both |

***Supplemental Table 3****.* ***Panel coverage original panel, MiSeq 2x151 bp***

*Panel coverage, original panel, sequenced on MiSeq. Coverage presented per gene, as percentage of bases covered above 30X and mean coverage (X), total as well as for exons, introns and UTRs separately. All values are mean values based on all sequenced samples.*

| **Gene** | **Total (%)** | **Total Mean (X)** | **Exons (%)** | **Exon Mean (X)** | **Introns (%)** | **Introns Mean (X)** | **UTR´s (%)** | **UTR´s Mean (X)** |
| --- | --- | --- | --- | --- | --- | --- | --- | --- |
| *ABCG5* | 95.7 | *157* | 100 | *194* | 95.2 | *153* | 100 | *170* |
| *ABCG8* | 95.8 | *168* | 100 | *188* | 95.1 | *168* | 99.3 | *164* |
| *APOB* | 99.5 | *168* | 100 | *157* | 99.2 | *173* | 100 | *151* |
| *APOE* | 99.0 | *225* | 100 | *263* | 98.5 | *208* | 100 | *257* |
| *LIPA* | 14.0 | *18* | 100 | *154* | 8.0 | *7* | 100 | *191* |
| *LDLR* | 85.7 | *141* | 100 | *248* | 87.7 | *138* | 42.2 | *88* |
| *LDLRAP1* | 99.2 | *227* | 100 | *239* | 99.1 | *227* | 100 | *226* |
| *PCSK9* | 100 | *227* | 100 | *247* | 100 | *226* | 100 | *218* |
| *SLCO1B1* | 85.9 | *108* | 99.4 | *125* | 85.5 | *108* | 100 | *116* |

***Supplemental Table 4****.* ***Panel coverage updated panel, NextSeq2000 2x301 bp***

*Panel coverage, updated panel, sequenced on NextSeq2000*. *Coverage presented per gene, as percentage of bases covered above 30X and mean coverage (X), total as well as for exons, introns and UTRs separately. All values are mean values based on all sequenced samples.*

| **Gene** | **Total (%)** | **Total Mean (X)** | **Exons (%)** | **Exon Mean (X)** | **Introns (%)** | **Introns Mean (X)** | **UTR´s (%)** | **UTR´s Mean (X)** |
| --- | --- | --- | --- | --- | --- | --- | --- | --- |
| *ABCG5* | 99.8 | *1032* | 100 | *1310* | 100 | *1004* | 100 | *1174* |
| *ABCG8* | 97.6 | *1120* | 100 | *1362* | 97.1 | *1115* | 100 | *1061* |
| *APOB* | 100 | *1246* | 100 | *1214* | 100 | *1265* | 100 | *1036* |
| *APOE* | 100 | *1332* | 100 | *1489* | 100 | *1286* | 100 | *1149* |
| *LIPA* | 24.6 | *132* | 100 | *980* | 19.4 | *59* | 100 | *1358* |
| *LDLR* | 97.5 | *887* | 100 | *1495* | 97.9 | *849* | 88.6 | *862* |
| *LDLRAP1* | 100 | *1599* | 100 | *1650* | 100 | *1606* | 100 | *1495* |
| *PCSK9* | 100 | *1596* | 100 | *1722* | 100 | *1592* | 100 | *1475* |
| *SLCO1B1* | 83.8 | *731* | 100 | *1009* | 83.4 | *724* | 100 | *918* |

***Supplemental Table 5****.* ***Coverage of included SNP markers***

*Coverage of included SNPs (polygenic risk markers and pharmacogenetic markers) for the original panel and the updated panel. Data is presented as average coverage (X) per position. Not all SNPs were included in the first design, only SNPs located within included genes therefore received coverage. All values are mean values based on all sequenced samples.*

| **RSID** | **Gene** | **Original panel, mean coverage (X)** | **Updated panel, mean coverage (X)** |
| --- | --- | --- | --- |
| **Markers for LDL-C polygenic risk score, 12 w-PRS** | | | |
| rs2479409 | *PCSK9* | - | 1063 |
| rs4299376 | *ABCG8* | 169 | 987 |
| rs1367117 | *APOB* | 220 | 1512 |
| rs6511720 | *LDLR* | 220 | 1527 |
| rs629301 | *CELSR2* | - | 1387 |
| rs429358 | *APOE* | 245 | 1631 |
| rs7412 | *APOE* | 233 | 1435 |
| rs1564348 | *SLC22A1* | - | 957 |
| rs1800562 | *HFE* | - | 1108 |
| rs3757354 | *MYLIP* | - | 1106 |
| rs11220462 | *ST3GAL4* | - | 1272 |
| rs8017377 | *NYNRIN* | - | 1082 |
| **Markers for statin clearance/statin response** | | | |
| rs4149056 | *SLCO1B1* | 120 | 986 |
| rs2231142 | *ABCG2* | 127 | 925 |

## ***Supplemental Table 6.*** ***Effect of fragment length and sequencing method.***

## *Increased fragment lengths and sequence read lengths resulted in longer insert sizes, improved mapping and higher on-target rates. Analysed on a subset of samples, n = 14, using the same extracted DNA, longer fragments with 2x301 bp paired-end reads resulted in longer insert sizes, higher percentage mapped reads and higher on-target rate compared to the approach with shorter fragments and 2x151bp reads.*

| **Sequencing method** | **Fragmentation approach** | **Insert size (bp)** | **Mapped reads (%)** | **On-target rate (%)** |
| --- | --- | --- | --- | --- |
| 2x151 bp | Shorter | 288 ± 23 | 99.3 | 23.4 |
| 2x301 bp | Longer | 323 ± 26 | 100 | 29.4 |

***Supplemental Table 7. Outcome of variant calling validation***

*Results of variant calling validation using truth set from Platinum genome presented both as a total for both SNVs/indels, and separately for SNVs and indels for both approaches (shorter fragments, shorter reads and original gene panel, and longer fragments, longer reads and updated gene panel). “Unfiltered” refers to all variants in confident regions, “Filtered” refers to confident variants in non-problematic regions. “Manual inspection” refers to remaining false positives and negatives after manual inspection of filtered variants in IGV and removal of variants located in poly-N regions or repeats i.e. the true number of false positive and negative variants with corresponding sensitivity and positive predictive value (PPV). “FN in relevant regions” refers to the number of false negatives not in* SCLO1B1 *introns.*

1. Number of variants detected from NA12878, shorter fragmentation during library preparation, hybridisation with original gene panel, and sequencing on MiSeq using 2x151 bp, compared to truth set from Platinum genome.

| **Variant type** | **Filtering steps** | **TP (n)** | **FP (n)** | **FN (n)** | **Sensitivity** | **PPV** |
| --- | --- | --- | --- | --- | --- | --- |
| Total | Unfiltered | 627 | 26 | 77 | 0.891 | 0.960 |
|  | Filtered | 527 | 14 | 17 | 0.969 | 0.974 |
|  | FN and FP after manual inspection | 527 | 0 | 9 | 0.983 | 1 |
|  | FN in relevant regions |  |  | 7 |  |  |
| SNVs | Unfiltered | 562 | 6 | 53 | 0.914 | 0.989 |
|  | Filtered | 485 | 5 | 15 | 0.970 | 0.990 |
|  | FN and FP after manual inspection | 485 | 0 | 8 | 0.984 | 1 |
|  | FN in relevant regions |  |  | 7 |  |  |
| indels | Unfiltered | 65 | 20 | 24 | 0.730 | 0.765 |
|  | Filtered | 42 | 9 | 2 | 0.955 | 0.824 |
|  | FN and FP after manual inspection | 42 | 0 | 1 | 0.977 | 1 |
|  | FN in relevant regions |  |  | 0 |  |  |

1. Number of variants detected from NA12878, longer fragmentation during library preparation, hybridisation with updated gene panel, and sequencing on NextSeq2000 using 2x301 bp, compared to truth set from Platinum genome. Here, variants are presented as the range of variants detected after four separate sample preparations with subsequent sequencing. Sensitivity and PPV are presented as average values showing little variation and high reproducibility. False negatives resulted as a lack of coverage in intronic regions of *SLCO1B1*.

| **Variant type** | **Filtering steps** | **TP (n)** | **FP (n)** | **FN (n)** | **Sensitivity** | **PPV** |
| --- | --- | --- | --- | --- | --- | --- |
| Total | Unfiltered | 639-646 | 28-35 | 58-65 | 0.915 | 0.956 |
|  | Filtered | 521-527 | 1-2 | 18-23 | 0.964 | 0.998 |
|  | FN and FP after manual inspection | 522-527 | 0 | 18-22 | 0.965 | 1 |
|  | FN in relevant regions |  |  | 0-1 |  |  |
| SNVs | Unfiltered | 562-571 | 2-6 | 44-53 | 0.925 | 0.996 |
|  | Filtered | 480-485 | 0-1 | 15-20 | 0.967 | 1 |
|  | FN and FP after manual inspection | 480-485 | 0 | 15-20 | 0.967 | 1 |
|  | FN in relevant regions |  |  | 0-1 | 0.997 |  |
| indels | Unfiltered | 65-76 | 20-33 | 13-24 | 0.843 | 0.730 |
|  | Filtered | 41-42 | 1 | 2-3 | 0.943 | 0.976 |
|  | FN and FP after manual inspection | 41-42 | 0 | 2-3 | 0.943 | 1 |
|  | FN in relevant regions |  |  | 0 |  |  |

***Supplemental Table 8.*** ***Variants of uncertain significance in non-coding regions of dominant FH-genes***

*Rare non-coding variants in LDLR, APOB and PCSK9 found in the study population. Variants are sorted per gene, location (intronic/UTR) and number of observations. All variants are heterozygous. The following PM2 thresholds for each gene were used to de-select frequently occurring variants in FH-related genes;* LDLR *0.2,* APOB *0.096,* APOE *0.05,* PCSK9 *0.192,* LDLRAP1 *0.05,* ABCG5 *0.212,* ABCG8 *0.282,* LIPA *0.15*

| ***Gene*** | ***Mode of inheritance*** | ***Location*** | ***c.position*** | ***Number*** |
| --- | --- | --- | --- | --- |
| *APOB* | Dominant | Intronic | c.82+37G>A | 3 |
| *APOB* | Dominant | Intronic | c.238-144A>G | 1 |
| *APOB* | Dominant | Intronic | c.384-763_384-760delAAGT | 1 |
| *APOB* | Dominant | Intronic | c.384-257C>T | 1 |
| *APOB* | Dominant | Intronic | c.693+325G>T | 1 |
| *APOB* | Dominant | Intronic | c.905-500C>A | 1 |
| *APOB* | Dominant | Intronic | c.1352+199C>G | 1 |
| *APOB* | Dominant | Intronic | c.1352+631T>G | 1 |
| *APOB* | Dominant | Intronic | c.1352+689T>C | 1 |
| *APOB* | Dominant | Intronic | c.1353-619T>C | 1 |
| *APOB* | Dominant | Intronic | c.2605-70G>C | 1 |
| *APOB* | Dominant | Intronic | c.2817-364G>C | 1 |
| *APOB* | Dominant | Intronic | c.3000-245T>A | 1 |
| *APOB* | Dominant | Intronic | c.3000-54C>T | 1 |
| *APOB* | Dominant | Intronic | c.3122-703G>A | 1 |
| *APOB* | Dominant | Intronic | c.3122-453A>G | 1 |
| *APOB* | Dominant | Intronic | c.3122-111T>C | 1 |
| *APOB* | Dominant | Intronic | c.3332+293C>A | 1 |
| *APOB* | Dominant | Intronic | c.3332+387delC | 1 |
| *APOB* | Dominant | Intronic | c.11788+121T>C | 1 |
| *APOB* | Dominant | Intronic | c.12088-73G>A | 1 |
| *LDLR* | Dominant | 3´UTR | c.*599G>A | 1 |
| *LDLR* | Dominant | Intronic | c.67+2291T>C | 1 |
| *LDLR* | Dominant | Intronic | c.67+2759T>G | 1 |
| *LDLR* | Dominant | Intronic | c.67+2912G>A | 1 |
| *LDLR* | Dominant | Intronic | c.67+3310A>G | 1 |
| *LDLR* | Dominant | Intronic | c.67+3699G>A | 1 |
| *LDLR* | Dominant | Intronic | c.67+4156C>T | 1 |
| *LDLR* | Dominant | Intronic | c.68-2523A>G | 1 |
| *LDLR* | Dominant | Intronic | c.68-3132delG | 1 |
| *LDLR* | Dominant | Intronic | c.68-655A>G | 1 |
| *LDLR* | Dominant | Intronic | c.68-720C>G | 1 |
| *LDLR* | Dominant | Intronic | c.190+101C>T | *2* |
| *LDLR* | Dominant | Intronic | c.191-390G>A | 1 |
| *LDLR* | Dominant | Intronic | c.313+531C>T | 1 |
| *LDLR* | Dominant | Intronic | c.695-461T>A | 1 |
| *LDLR* | Dominant | Intronic | c.817+173C>T | 1 |
| *LDLR* | Dominant | Intronic | c.940+232T>C | 1 |
| *LDLR* | Dominant | Intronic | c.941-1184C>T | 2 |
| *LDLR* | Dominant | Intronic | c.1060+245C>A | 1 |
| *LDLR* | Dominant | Intronic | c.1060+33C>G | 1 |
| *LDLR* | Dominant | Intronic | c.1186+656G>A | 1 |
| *LDLR* | Dominant | Intronic | c.1186+762G>A | 1 |
| *LDLR* | Dominant | Intronic | c.1586+386A>G | 1 |
| *LDLR* | Dominant | Intronic | c.1587-461A>G | 1 |
| *LDLR* | Dominant | Intronic | c.1587-475C>T | 1 |
| *LDLR* | Dominant | Intronic | c.1845+590C>G | 1 |
| *LDLR* | Dominant | Intronic | c.1846-476C>T | 1 |
| *LDLR* | Dominant | Intronic | c.1846-54G>C | 1 |
| *LDLR* | Dominant | Intronic | c.1987+21C>T | 1 |
| *LDLR* | Dominant | Intronic | c.2140+1103G>A | 1 |
| *LDLR* | Dominant | Intronic | c.2311+1203A>T | 1 |
| *LDLR* | Dominant | Intronic | c.2312-1978C>G | 1 |
| *LDLR* | Dominant | Intronic | c.2312-28G>A | 1 |
| *LDLR* | Dominant | Intronic | c.2547+439T>C | 1 |
| *PCSK9* | Dominant | 3´UTR | c.*1097C>T | 1 |
| *PCSK9* | Dominant | 3´UTR | c.*887C>T | 1 |
| *PCSK9* | Dominant | Intronic | c.208-1539G>A | 2 |
| *PCSK9* | Dominant | Intronic | c.400-172C>T | 2 |
| *PCSK9* | Dominant | Intronic | c.1504-109C>G | 1 |
| *PCSK9* | Dominant | Intronic | c.1681+64G>T | 1 |
| *PCSK9* | Dominant | Intronic | c.1682-789C>T | 1 |
| *PCSK9* | Dominant | Intronic | c.1682-824C>T | 1 |
| *PCSK9* | Dominant | Intronic | c.1864-245dupT | 1 |
| *PCSK9* | Dominant | Intronic | c.207+920C>A | 1 |
| *PCSK9* | Dominant | Intronic | c.399+1046C>T | 1 |
| *PCSK9* | Dominant | Intronic | c.399+212C>T | 1 |
| *PCSK9* | Dominant | Intronic | c.399+693C>T | 1 |
| *PCSK9* | Dominant | Intronic | c.523+2764C>T | 1 |
| *PCSK9* | Dominant | Intronic | c.524-1612C>G | 1 |
| *PCSK9* | Dominant | Intronic | c.799+702C>A | 1 |
| *PCSK9* | Dominant | Intronic | c.800-1274C>T | 1 |
| *PCSK9* | Dominant | Intronic | c.800-134C>A | 1 |
| *PCSK9* | Dominant | Intronic | c.800-3C>T | 1 |
| *PCSK9* | Dominant | Intronic | c.996+160C>A | 1 |

***Supplemental Table 9.*** ***Variants of uncertain significance in non-coding regions of recessive FH-genes***

*Rare non-coding variants in* ABCG8*,* LDLRAP1 *and* LIPA *found in the study population. Variants are sorted per gene, location (intronic/UTR) and number of observations. All variants are heterozygous. The following PM2 thresholds for each gene were used to de-select frequently occurring variants in FH-related genes;* LDLR *0.2,* APOB *0.096,* APOE *0.05,* PCSK9 *0.192,* LDLRAP1 *0.05,* ABCG5 *0.212,* ABCG8 *0.282,* LIPA *0.15*

| ***Gene*** | ***Mode of inheritance*** | ***Location*** | ***c.position*** | ***Number*** |
| --- | --- | --- | --- | --- |
| *ABCG5* | Recessive | Intronic | c.266-2684C>T | 1 |
| *ABCG5* | Recessive | Intronic | c.502-1662C>G | 1 |
| *ABCG5* | Recessive | Intronic | c.774+423A>C | 1 |
| *ABCG8* | Recessive | 3´UTR | c.*1543G>T | 1 |
| *ABCG8* | Recessive | 3´UTR | c.*3248A>G | 1 |
| *ABCG8* | Recessive | 3´UTR | c.*612C>T | 1 |
| *ABCG8* | Recessive | Intronic | c.322+221C>A | 1 |
| *ABCG8* | Recessive | Intronic | c.64-2402A>G | 1 |
| *ABCG8* | Recessive | Intronic | c.-915C>T | 1 |
| *ABCG8* | Recessive | Intronic | c.964+1328C>T | 1 |
| *ABCG8* | Recessive | Intronic | c.964+4509G>A | 1 |
| *ABCG8* | Recessive | Intronic | c.964+6321C>G | 1 |
| *ABCG8* | Recessive | Intronic | c.964+9288A>C | 1 |
| *LDLRAP1* | Recessive | 3´UTR | c.*31C>G | 1 |
| *LDLRAP1* | Recessive | Intronic | c.460-775G>A | 1 |
| *LDLRAP1* | Recessive | Intronic | c.782+307A>G | 1 |
| *LDLRAP1* | Recessive | Intronic | c.88+3213G>A | 1 |
| *LDLRAP1* | Recessive | Intronic | c.89-2008delCAGT | 1 |
| *LDLRAP1* | Recessive | Intronic | c.89-2404T>C | 1 |
| *LDLRAP1* | Recessive | Intronic | c.99+1265G>A | 1 |
| *LIPA* | Recessive | 3´UTR | c.*891G>A | 1 |

***Supplemental Table 10. Pharmacogenetic markers***

*Number of screened individuals (n=133) with the rs41419056 variant in* SLCO1B1 *and/or the rs2231142 variant in* ABCG2*.*

| **SNP ID** | **Gene** | **Allele** | **Allele frequency** | **Number of individuals** | **Genotype** | **Frequency of genotypes (%)** |
| --- | --- | --- | --- | --- | --- | --- |
| rs41419056 | *SLCO1B1* | T | 0.8722 | 102 | T/T | 76.6 |
|  |  | C | 0.1278 | 31 | C/T, C/C | 23.3 |
| rs2231142 | *ABCG2* | G | 0.8646 | 98 | G/G | 73.6 |
|  |  | T | 0.1353 | 35 | G/T, T/T | 26.3 |


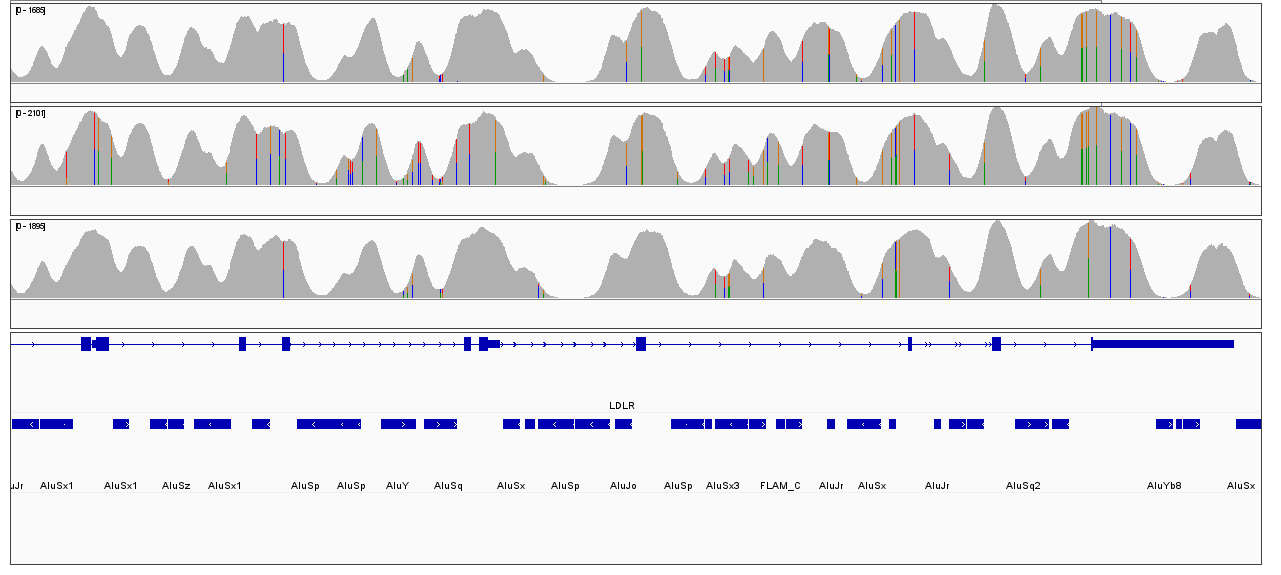


***Supplemental Figure 1. Panel characteristics.***

*IGV screenshot of parts of LDLR, showing intronic regions without coverage for three selected samples hybridised with the updated version of the gene panel. Coverage is high, but variable, and Alu-repeats (blue track) results in reduced or absent coverage in intron 14 and 3´UTR, indicated by arrows in the figure.*


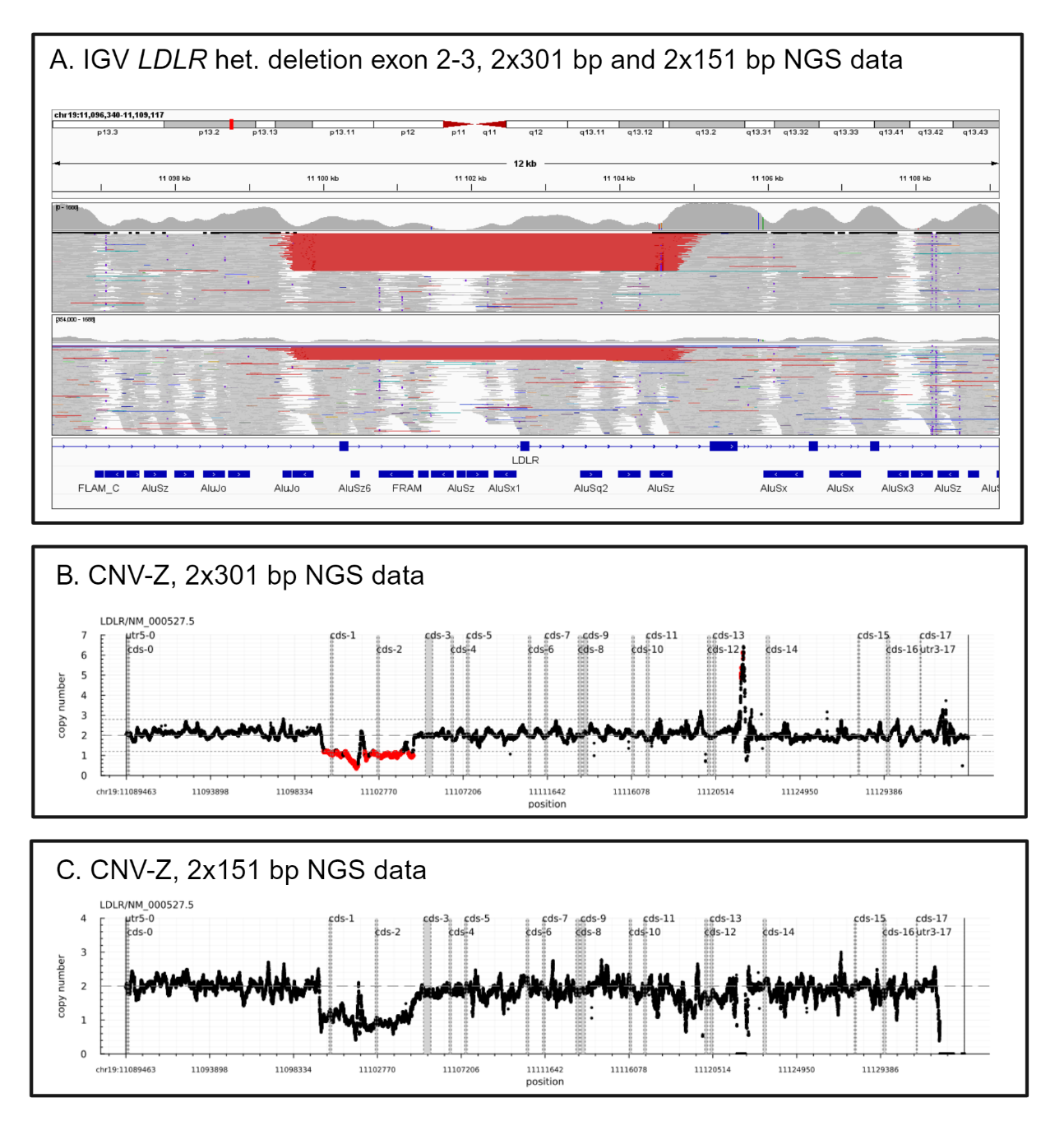


***Supplemental Figure 2.* *Effect of longer insert sizes on CNV calling using CNV-Z.***

*A) IGV graph displaying a heterozygous deletion of exons 2-3, with visible breakpoints in IGV. The same sample has been sequenced twice, both with 2x301 bp read length and with 2x151 bp read length. Split reads are displayed in red, indicating a deletion spanning exon 2 and exon 3, with visible breakpoints. CNV-Z detects the copy number variant when NGS reads were obtained with 2x301 bp reads and longer fragments (B). When using reads from 2x151 bp sequencing and shorter fragments, the deletion was not detected with confidence, although indicated as can be seen in C.*

# References

1. Eberle, M.A., et al., *A reference data set of 5.4 million phased human variants validated by genetic inheritance from sequencing a three-generation 17-member pedigree.* Genome Research, 2017. **27**(1): p. 157-164.DOI: 10.1101/gr.210500.116.

2. Li, H., *Aligning sequence reads, clone sequences and assembly contigs with BWA-MEM*. 2013: arXiv:1303.3997v1 [q-bio.GN], <https://github.com/lh3/bwa>.

3. Li, H., et al., *The Sequence Alignment/Map format and SAMtools.* Bioinformatics, 2009. **25**(16): p. 2078-9.DOI: 10.1093/bioinformatics/btp352.

4. Van der Auwera, G.A., et al., *From FastQ data to high confidence variant calls: the Genome Analysis Toolkit best practices pipeline.* Curr Protoc Bioinformatics, 2013. **43**(1110): p. 11.10.1-11.10.33.DOI: 10.1002/0471250953.bi1110s43.

5. Danecek, P., et al., *Twelve years of SAMtools and BCFtools.* Gigascience, 2021. **10**(2).DOI: 10.1093/gigascience/giab008.

6. Erik Garrison, G.M., *Haplotype-based variant detection from short-read sequencing*. 2012, arXiv:1207.3907v2 [q-bio.GN].

7. Danecek, P., et al., *The variant call format and VCFtools.* Bioinformatics, 2011. **27**(15): p. 2156-2158.DOI: 10.1093/bioinformatics/btr330.

8. Philip Ewels, M.M., Sverker Lundin, Max Käller, *MultiQC: Summarize analysis results for multiple tools and samples in a single report.* Bioinformatics, 2016. 10.1093/bioinformatics/btw354.DOI: 10.1093/bioinformatics/btw354.

9. Adolfsson, E., et al., *CNV-Z; a new tool for detecting copy number variation in next generation sequencing data.* SoftwareX, 2023. **24**: p. 101530.DOI: <https://doi.org/10.1016/j.softx.2023.101530>.

10. Krusche, P., et al., *Best practices for benchmarking germline small-variant calls in human genomes.* Nature Biotechnology, 2019. **37**(5): p. 555-560.DOI: 10.1038/s41587-019-0054-x.

11. Mandelker, D., et al., *Navigating highly homologous genes in a molecular diagnostic setting: a resource for clinical next-generation sequencing.* Genetics in Medicine, 2016. **18**(12): p. 1282-1289.DOI: <https://doi.org/10.1038/gim.2016.58>.

12. Robinson, J.T., et al., *Integrative genomics viewer.* Nat Biotechnol, 2011. **29**(1): p. 24-6.DOI: 10.1038/nbt.1754.

13. Talmud, P.J., et al., *Use of low-density lipoprotein cholesterol gene score to distinguish patients with polygenic and monogenic familial hypercholesterolaemia: a case-control study.* Lancet, 2013. **381**(9874): p. 1293-301.DOI: 10.1016/s0140-6736(12)62127-8.
